# *catena*-Poly[[{bis­[tetra­aqua­(2-hy­droxy-3,4-dioxocyclo­but-1-en-1-olato-κ*O* ^1^)bariumstrontium(0.35/0.65)]di-μ-aqua}­bis­(μ-2-hy­droxy-4-oxocyclo­but-1-ene-1,3-diolato-κ^2^ *O* ^1^:*O* ^3^)] monohydrate]

**DOI:** 10.1107/S1600536811002996

**Published:** 2011-01-29

**Authors:** Chahrazed Trifa, Amira Bouhali, Sofiane Bouacida, Chaouki Boudaren, Thierry Bataille

**Affiliations:** a Unité de Recherche de Chimie de l’Environnement et Moléculaire Structurale, CHEMS, Université Mentouri–Constantine, 25000 Algeria; b Département Sciences de la Matière, Faculté des Sciences Exactes et Sciences de la Nature et de la Vie, Université Larbi Ben M’hidi, Oum El Bouaghi, Algeria; c Sciences Chimiques de Rennes, UMR 6226 CNRS – Université de Rennes 1, Avenue du Général Leclerc, 35042 Rennes Cedex, France

## Abstract

The title structure, {[Ba_0.71_Sr_1.29_(C_4_HO_4_)_4_(H_2_O)_10_]·H_2_O}_*n*_, is built from dimers of edge-sharing monocapped square anti­prisms [(Ba/Sr)O_3_(H_2_O)_6_], in which barium and strontium are statistically disordered [ratio 0.353 (8):0.647 (8)] on the same crystallographic site. Such dimers are connected *via* bidentate hydrogen squarate groups [HC_4_O_4_]^−^, leading to chains that propagate along the *b* axis. Inter- and intra­molecular O—H⋯O hydrogen bonds maintain the crystal packing through a three-dimensional network.

## Related literature

For related transition metal squarate structures, see: West & Niu (1963 ▶); Lee *et al.* (1996 ▶); Haben-Schuss & Gerstein (1974 ▶). For related alkaline earth squarate structures, see: Robl & Weis (1986 ▶, 1987 ▶); Robl *et al.* (1987 ▶); Bouayad *et al.* (1995 ▶). For related rare earth squarate structures, see: Trombe *et al.* (1988 ▶, 1990 ▶, 1991 ▶); Bénard-Rocherullé & Akkari (2005 ▶). For the first synthesis of squaric acid (3,4-dihy­droxy­cyclo­but-3-ene-1,2-dione), see: Cohen *et al.* (1959 ▶).
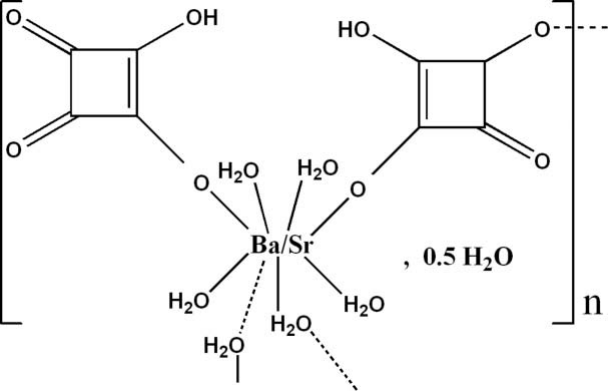


## Experimental

### 

#### Crystal data

[Ba_0.71_Sr_1.29_(C_4_HO_4_)_4_(H_2_O)_10_]·H_2_O
*M* *_r_* = 860.70
Monoclinic, 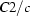

*a* = 25.3592 (9) Å
*b* = 8.8993 (3) Å
*c* = 14.1286 (5) Å
β = 119.974 (2)°
*V* = 2762.07 (18) Å^3^
*Z* = 4
Mo *K*α radiation
μ = 3.62 mm^−1^
*T* = 295 K
0.09 × 0.08 × 0.08 mm

#### Data collection

Nonius KappaCCD diffractometer
18241 measured reflections
3173 independent reflections
2616 reflections with *I* > 2σ(*I*)
*R*_int_ = 0.077

#### Refinement

*R*[*F* ^2^ > 2σ(*F* ^2^)] = 0.033
*wR*(*F* ^2^) = 0.088
*S* = 1.06
3173 reflections
239 parameters
4 restraints
H atoms treated by a mixture of independent and constrained refinement
Δρ_max_ = 0.96 e Å^−3^
Δρ_min_ = −1.02 e Å^−3^

### 

Data collection: *COLLECT* (Nonius, 1998 ▶); cell refinement: *SCALEPACK* (Otwinowski & Minor, 1997 ▶); data reduction: *DENZO* (Otwinowski & Minor, 1997 ▶) and *SCALEPACK*; program(s) used to solve structure: *SIR2002* (Burla *et al.*, 2003 ▶); program(s) used to refine structure: *SHELXL97* (Sheldrick, 2008 ▶); molecular graphics: *DIAMOND* (Brandenburg & Berndt, 2001 ▶); software used to prepare material for publication: *WinGX* (Farrugia, 1999 ▶).

## Supplementary Material

Crystal structure: contains datablocks global, I. DOI: 10.1107/S1600536811002996/bq2274sup1.cif

Structure factors: contains datablocks I. DOI: 10.1107/S1600536811002996/bq2274Isup2.hkl

Additional supplementary materials:  crystallographic information; 3D view; checkCIF report

## Figures and Tables

**Table 1:** Selected bond lengths (Å)

Ba1—O1	2.728 (2)
Ba1—O5	2.688 (2)
Ba1—O9*W*	2.729 (3)
Ba1—O10*W*	2.772 (3)
Ba1—O11*W*	2.722 (3)
Ba1—O12*W*	2.644 (3)
Ba1—O13*W*	2.782 (3)
Ba1—O3^i^	2.6720 (19)
Ba1—O10*W*^ii^	2.786 (2)

Symmetry codes: (i) 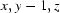; (ii) 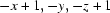.

**Table 2:** Hydrogen-bond geometry (Å, °)

*D*—H⋯*A*	*D*—H	H⋯*A*	*D*⋯*A*	*D*—H⋯*A*
O1*W*—H1*W*⋯O2^ii^	0.82 (4)	2.55 (4)	2.874 (2)	105 (3)
O1*W*—H1*W*⋯O7^iii^	0.82 (4)	2.47 (4)	3.193 (3)	148 (4)
O4—H4⋯O5^iv^	0.82	1.79	2.603 (3)	171
O8—H8⋯O1	0.82	1.77	2.575 (3)	169
O9*W*—H9*A*⋯O1*W*^iv^	0.84 (3)	2.47 (3)	3.197 (5)	147 (4)
O9*W*—H9*B*⋯O3^v^	0.87 (4)	1.97 (4)	2.821 (3)	167 (5)
O10*W*—H10*A*⋯O11*W*^vi^	0.78 (5)	2.54 (4)	3.150 (4)	137 (4)
O10*W*—H10*B*⋯O2^ii^	0.86 (4)	1.88 (4)	2.711 (4)	164 (4)
O11*W*—H11*A*⋯O1*W*	0.86 (3)	2.06 (4)	2.880 (5)	159 (4)
O11*W*—H11*B*⋯O6^vii^	0.87 (4)	1.91 (4)	2.777 (3)	176 (5)
O12*W*—H12*A*⋯O13*W*^viii^	0.83 (4)	2.00 (4)	2.803 (3)	164 (4)
O12*W*—H12*B*⋯O7^ix^	0.84 (4)	1.87 (4)	2.711 (3)	178 (5)
O13*W*—H13*A*⋯O6^x^	0.86 (5)	2.00 (4)	2.736 (3)	143 (3)
O13*W*—H13*B*⋯O8^xi^	0.84 (4)	2.24 (4)	3.014 (3)	154 (4)

Symmetry codes: (ii) 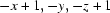; (iii) 
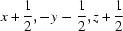
; (iv) 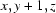; (v) 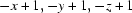; (vi) 
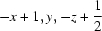
; (vii) 
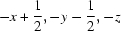
; (viii) 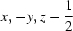; (ix) 
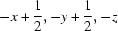
; (x) 
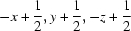
; (xi) 
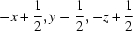
.

**Fig. 1.**
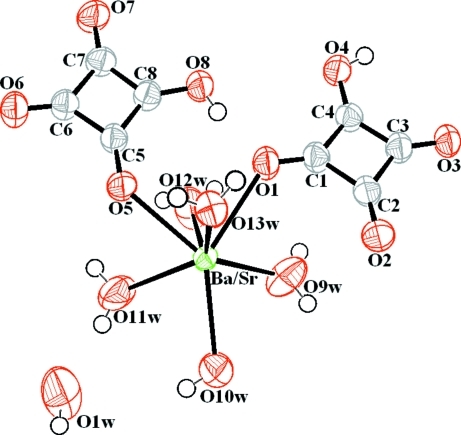
An ORTEP-3 (Farrugia, 1997) drawing of (I), with the atom-numbering scheme. Displacement ellipsoids are drawn at the 50% probability level.

**Fig. 2.**
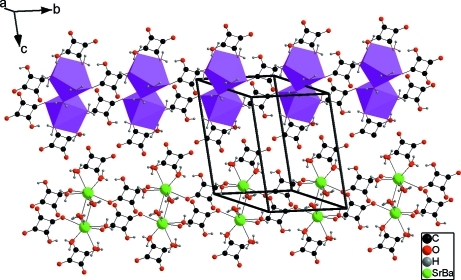
A packing diagram of (I), showing the chains.

## supplementary crystallographic information

### Comment

Squaric acid, (3,4-dihydroxycyclobut-3-ene-1,2-dione, H_2_C_4_O_4_, H_2_sq), synthesized for the first time by Cohen *et al.* (1959), has been of interest because of its cyclic structure and its possible aromaticity. (West *et al.*, 1963) have described the preparation from aqueous solutions of 'isostructural' divalent metal squarates of general formula MC_4_O_4_.2H_2_O, and predicted a chelated linear polymer structure, the structure of Ni(C_4_O_4_).2H_2_O being reported later by (Haben-schuss *et al.*, 1974). In the structurally well understood metal squarates *M*(C_4_O_4_)(H_2_O)_4_ (*M* = Mn, Fe,Co, Ni, Zn) (Lee *et al.*, 1996), the C_4_O_4_^2-^ entity serves as a bridging ligand between two metal ions (µ-2) in *trans* positions while it acts as a fourfold monodentate (µ-4) ligand between metals in the three-dimensional polymeric structures of *M*(C_4_O_4_)(H_2_O)_2_ (*M* = Mn, Fe, Co, Ni, Cu, Zn). However, the cyclic group only chelate the largest cations such as alkaline earth (Robl & Weis, 1986, 1987; Robl *et al.*, 1987; Bouayad *et al.*, 1995) or rare earth elements (Trombe *et al.*, 1988; Trombe *et al.*, 1990; Trombe *et al.*, 1991; Bérnard-Rocherullé & Akkari, 2005).

Surprisingly, the crystal structures of barium squarate hydrate and strontium squarate hydrate are rather different. It is of interest to explore the possibilities of mixing cations, to obtain new compounds or solid solutions. Here, we synthesized hemi hydrate barium strontium squarate, [Ba0.35 Sr0.65 (HC_4_O_4_)_2_ (H_2_O)_5_] 0.5 H_2_O, for which the Ba / Sr ratio has been determined from EDX and single-crystal diffraction data.

The asymmetric unit contains two metal atoms, two hydrogen squarate anions, five aqua ligands and half water molecule. The Barium and Strontium ions are disordered on the same site as well as a solvent water molecule situated on the twofold axis at (4 e; 0, *y*, 1/4).

The structure is formed from chains, bridged by the hydrogeno squarate group acting as a bidentate ligand in a *trans* position (Fig. 2). The three-dimensionality is ensured by a strong O—H···O hydrogen bond (Table 1. The free water molecule is sandwiched between these chains.

### Experimental

All chemical were commercially available and used as received. For convenience, 3,4-dihydroxycyclobut-3-ene-1,2-dione (H_2_C_4_O_4_) is named squaric acid hereafter. Typically, Poly [pentaaqua di squarato barium strontium] hemi hydrate was synthesized by hydrothermal reaction starting from a mixture of barium chloride BaCl_2_, 2H_2_O (2 mmol), strontium chloride SrCl_2_, 6H2O (2 mmol) squaric acid H_2_C_4_O_4_, oxalic acid H_2_C_2_O_4_, 2H2O (1 mmol) and water (4 ml). The whole was stirred for 30 minutes until homogeneous. The final mixture was sealed in a 23 ml Teflon-lined acid digestion bomb (Parr) and heated at 423 K for 48 h under autogeneous pressure and then cooled down to room temperature. The yellow crystalline product obtained were collected by filtration, thoroughly washed with distilled water and ethanol, and finally dried at room temperature. The chemical formula was derived from the Ba/Sr ratio (1/2) obtained by energy dispersive X-ray spectrometry (EDX), and from the crystal structure determination reported below.

### Refinement

All H atoms were localized on Fourier maps and refined isotropically, except for H atoms for hydroxy groups of hydrogenosquarate (H4 and H8) which were introduced in calculated positions and treated as riding on their parent O atom (with O—H = 0.82Å and *U*_iso_(H) =1.5*U*_eq_(O)). Some distances (O—H)of coordinate water molecule are refined with soft constraints, the O—H distances is restrained to 0.85 Å. (O11W—H11A, O9W—H9A and O1W—H1W).

### Crystal data

**Table:**

[Ba_0.71_Sr_1.29_(C_4_HO_4_)_4_(H_2_O)_10_]·H_2_O	*F*(000) = 1706.4
*M**_r_* = 860.70	*D*_x_ = 2.070 Mg m^−^^3^
Monoclinic, *C*2/*c*	Mo *K*α radiation, λ = 0.71073 Å
Hall symbol: -C 2yc	Cell parameters from 18241 reflections
*a* = 25.3592 (9) Å	θ = 2.9–27.5°
*b* = 8.8993 (3) Å	µ = 3.62 mm^−^^1^
*c* = 14.1286 (5) Å	*T* = 295 K
β = 119.974 (2)°	Cube, yellow
*V* = 2762.07 (18) Å^3^	0.09 × 0.08 × 0.08 mm
*Z* = 4	

### Data collection

**Table:**

Nonius KappaCCD diffractometer	*R*_int_ = 0.077
CCD rotation images, thick slices scans	θ_max_ = 27.5°, θ_min_ = 3.2°
18241 measured reflections	*h* = −32→32
3173 independent reflections	*k* = −10→11
2616 reflections with *I* > 2σ(*I*)	*l* = −18→18

### Refinement

**Table:**

Refinement on *F*^2^	H atoms treated by a mixture of independent and constrained refinement
Least-squares matrix: full	*w* = 1/[σ^2^(*F*_o_^2^) + (0.0418*P*)^2^ + 2.6738*P*] where *P* = (*F*_o_^2^ + 2*F*_c_^2^)/3
*R*[*F*^2^ > 2σ(*F*^2^)] = 0.033	(Δ/σ)_max_ = 0.004
*wR*(*F*^2^) = 0.088	Δρ_max_ = 0.96 e Å^−^^3^
*S* = 1.06	Δρ_min_ = −1.02 e Å^−^^3^
3173 reflections	Extinction correction: *SHELXL97* (Sheldrick, 2008), Fc^*^=kFc[1+0.001xFc^2^λ^3^/sin(2θ)]^-1/4^
239 parameters	Extinction coefficient: 0.0016 (2)
4 restraints	

### Special details

**Table:**

Geometry. Bond distances, angles *etc*. have been calculated using the rounded fractional coordinates. All su's are estimated from the variances of the (full) variance-covariance matrix. The cell e.s.d.'s are taken into account in the estimation of distances, angles and torsion angles

### Fractional atomic coordinates and isotropic or equivalent isotropic displacement parameters (Å^2^)

**Table:**

	*x*	*y*	*z*	*U*_iso_*/*U*_eq_	Occ. (<1)
C1	0.36573 (13)	0.3970 (3)	0.3325 (2)	0.0289 (6)	
C2	0.41697 (13)	0.4566 (3)	0.4371 (2)	0.0316 (6)	
C3	0.39687 (13)	0.6124 (3)	0.3940 (2)	0.0306 (6)	
C4	0.34755 (13)	0.5476 (3)	0.2960 (2)	0.0283 (6)	
C5	0.25484 (12)	−0.0504 (3)	0.1164 (2)	0.0271 (6)	
C6	0.20204 (13)	−0.1154 (3)	0.0196 (2)	0.0285 (6)	
C7	0.17992 (13)	0.0393 (3)	−0.0209 (2)	0.0306 (6)	
C8	0.23261 (13)	0.0965 (3)	0.0768 (2)	0.0292 (6)	
O1	0.34685 (9)	0.2666 (2)	0.29723 (16)	0.0340 (5)	
O2	0.45673 (10)	0.4025 (2)	0.52317 (17)	0.0443 (6)	
O1W	0.5	−0.4640 (6)	0.25	0.089	
O3	0.41410 (10)	0.7405 (2)	0.42950 (17)	0.0382 (5)	
O4	0.30067 (9)	0.6011 (2)	0.20752 (16)	0.0363 (5)	
H4	0.3002	0.6929	0.2116	0.055*	
O5	0.29950 (9)	−0.1065 (2)	0.20118 (15)	0.0338 (5)	
O6	0.18250 (10)	−0.2429 (2)	−0.01205 (16)	0.0360 (5)	
O7	0.13455 (10)	0.0922 (2)	−0.10122 (17)	0.0414 (5)	
O8	0.24809 (10)	0.2349 (2)	0.11254 (17)	0.0382 (5)	
H8	0.2789	0.2336	0.1729	0.057*	
O9W	0.48959 (13)	0.2259 (3)	0.3538 (2)	0.0565 (7)	
O10W	0.52933 (11)	−0.1012 (3)	0.4460 (2)	0.0431 (6)	
O11W	0.42281 (12)	−0.2067 (3)	0.2105 (2)	0.0590 (8)	
O12W	0.38389 (14)	0.1093 (3)	0.1430 (2)	0.0563 (7)	
O13W	0.33586 (12)	−0.0026 (2)	0.4289 (2)	0.0374 (6)	
Sr1	0.410752 (9)	0.005151 (19)	0.337327 (15)	0.02353 (12)	0.647 (8)
Ba1	0.410752 (9)	0.005151 (19)	0.337327 (15)	0.02353 (12)	0.353 (8)
H1W	0.5265 (15)	−0.516 (3)	0.298 (3)	0.05*	
H9A	0.4765 (16)	0.306 (3)	0.319 (3)	0.05*	
H9B	0.5230 (19)	0.233 (4)	0.416 (3)	0.05*	
H10A	0.5449 (18)	−0.078 (5)	0.413 (3)	0.05*	
H10B	0.5316 (18)	−0.197 (5)	0.443 (3)	0.05*	
H11A	0.4449 (16)	−0.286 (3)	0.237 (3)	0.05*	
H11B	0.3907 (19)	−0.220 (5)	0.147 (3)	0.05*	
H12A	0.3669 (18)	0.064 (5)	0.084 (3)	0.05*	
H12B	0.3786 (18)	0.202 (5)	0.132 (3)	0.05*	
H13A	0.3201 (18)	0.084 (5)	0.424 (3)	0.05*	
H13B	0.3087 (19)	−0.067 (4)	0.396 (3)	0.05*	

### Atomic displacement parameters (Å^2^)

**Table:**

	*U*^11^	*U*^22^	*U*^33^	*U*^12^	*U*^13^	*U*^23^
C1	0.0338 (15)	0.0260 (14)	0.0249 (13)	−0.0003 (11)	0.0131 (12)	−0.0017 (10)
C2	0.0354 (16)	0.0259 (13)	0.0279 (14)	0.0017 (12)	0.0116 (13)	−0.0019 (11)
C3	0.0335 (15)	0.0255 (14)	0.0290 (14)	−0.0012 (11)	0.0127 (13)	−0.0016 (11)
C4	0.0318 (14)	0.0242 (13)	0.0263 (13)	0.0012 (11)	0.0126 (12)	0.0012 (11)
C5	0.0287 (14)	0.0253 (13)	0.0256 (13)	−0.0012 (11)	0.0124 (11)	−0.0019 (10)
C6	0.0313 (15)	0.0276 (14)	0.0229 (13)	−0.0020 (11)	0.0107 (12)	0.0016 (10)
C7	0.0313 (15)	0.0304 (13)	0.0263 (14)	0.0006 (12)	0.0115 (12)	0.0037 (11)
C8	0.0307 (15)	0.0278 (14)	0.0268 (13)	−0.0021 (11)	0.0126 (12)	0.0001 (11)
O1	0.0396 (12)	0.0240 (10)	0.0297 (11)	−0.0027 (8)	0.0109 (9)	−0.0018 (7)
O2	0.0470 (13)	0.0351 (12)	0.0297 (11)	0.0089 (10)	0.0033 (10)	0.0010 (9)
O1W	0.147	0.056	0.032	0	0.02	0
O3	0.0419 (13)	0.0241 (10)	0.0354 (11)	0.0001 (8)	0.0093 (10)	−0.0042 (8)
O4	0.0363 (11)	0.0258 (10)	0.0313 (11)	0.0007 (9)	0.0051 (9)	0.0007 (8)
O5	0.0336 (11)	0.0275 (10)	0.0259 (10)	0.0022 (8)	0.0040 (9)	0.0023 (8)
O6	0.0433 (13)	0.0274 (10)	0.0287 (11)	−0.0059 (8)	0.0114 (10)	−0.0003 (8)
O7	0.0369 (12)	0.0364 (12)	0.0314 (11)	0.0010 (9)	0.0023 (10)	0.0061 (9)
O8	0.0370 (12)	0.0254 (10)	0.0350 (12)	0.0009 (8)	0.0050 (10)	−0.0017 (8)
O9W	0.0461 (15)	0.073 (2)	0.0362 (14)	−0.0110 (14)	0.0100 (12)	0.0038 (12)
O10W	0.0410 (13)	0.0407 (13)	0.0470 (14)	0.0010 (10)	0.0215 (11)	−0.0042 (11)
O11W	0.0410 (14)	0.073 (2)	0.0435 (15)	0.0081 (13)	0.0068 (12)	−0.0231 (14)
O12W	0.083 (2)	0.0384 (14)	0.0344 (13)	−0.0009 (13)	0.0191 (14)	0.0022 (10)
O13W	0.0392 (13)	0.0308 (13)	0.0392 (13)	−0.0006 (8)	0.0174 (11)	−0.0031 (8)
Sr1	0.02376 (16)	0.02152 (16)	0.01978 (15)	0.00041 (7)	0.00672 (10)	−0.00061 (7)
Ba1	0.02376 (16)	0.02152 (16)	0.01978 (15)	0.00041 (7)	0.00672 (10)	−0.00061 (7)

### Geometric parameters (Å, °)

**Table:**

Ba1—O1	2.728 (2)	O7—C7	1.236 (4)
Ba1—O5	2.688 (2)	O8—C8	1.315 (3)
Ba1—O9W	2.729 (3)	O4—H4	0.8200
Ba1—O10W	2.772 (3)	O8—H8	0.8200
Ba1—O11W	2.722 (3)	O9W—H9A	0.84 (3)
Ba1—O12W	2.644 (3)	O9W—H9B	0.87 (4)
Ba1—O13W	2.782 (3)	O10W—H10B	0.86 (4)
Ba1—O3^i^	2.6720 (19)	O10W—H10A	0.78 (5)
Ba1—O10W^ii^	2.786 (2)	O11W—H11B	0.87 (4)
Sr1—O1	2.728 (2)	O11W—H11A	0.86 (3)
Sr1—O5	2.688 (2)	O12W—H12A	0.83 (4)
Sr1—O9W	2.729 (3)	O12W—H12B	0.84 (4)
Sr1—O10W	2.772 (3)	O13W—H13A	0.86 (5)
Sr1—O11W	2.722 (3)	O13W—H13B	0.84 (4)
Sr1—O12W	2.644 (3)	O1W—H1W	0.82 (4)
Sr1—O13W	2.782 (3)	O1W—H1W^iii^	0.82 (3)
Sr1—O3^i^	2.6720 (19)	C1—C2	1.495 (4)
Sr1—O10W^ii^	2.786 (2)	C1—C4	1.427 (4)
O1—C1	1.259 (3)	C2—C3	1.498 (4)
O2—C2	1.226 (3)	C3—C4	1.444 (4)
O3—C3	1.234 (3)	C5—C8	1.423 (4)
O4—C4	1.311 (3)	C5—C6	1.473 (4)
O5—C5	1.269 (3)	C6—C7	1.490 (4)
O6—C6	1.230 (3)	C7—C8	1.451 (4)
			
O1···O4	3.218 (3)	O3···H9B^viii^	1.97 (4)
O1···O8	2.575 (3)	O4···H13B^xii^	2.83 (5)
O1···O12W	3.104 (4)	O4···H13A^xii^	2.68 (5)
O1···O13W	3.130 (3)	O5···H4^i^	1.7900
O1···C8	3.372 (3)	O5···H13B	2.67 (4)
O1W···O11W^iii^	2.880 (5)	O6···H13A^xiii^	2.00 (4)
O1W···O9W^iv^	3.197 (5)	O6···H11B^vi^	1.91 (4)
O1W···O2^ii^	2.874 (2)	O7···H1W^xiv^	2.47 (4)
O1W···O2^v^	2.874 (2)	O7···H12B^xi^	1.87 (4)
O1W···O7^vi^	3.193 (3)	O8···H13B^xii^	2.24 (4)
O1W···O7^vii^	3.193 (3)	O9W···H1W^x^	2.74 (3)
O1W···O11W	2.880 (5)	O11W···H10A^iii^	2.54 (5)
O1W···O9W^i^	3.197 (5)	O11W···H12A	2.91 (4)
O2···C3^viii^	3.291 (4)	O12W···H10A^iii^	2.85 (5)
O2···C2^viii^	3.217 (4)	O13W···H12A^ix^	2.00 (4)
O2···O1W^ix^	2.874 (2)	O13W···H10A^ii^	2.81 (4)
O2···O10W^ii^	2.711 (4)	C1···O8	3.373 (3)
O2···O1W^ii^	2.874 (2)	C1···C5^xii^	3.506 (5)
O2···O2^viii^	3.113 (4)	C1···O7^xi^	3.266 (4)
O3···O13W^x^	3.024 (3)	C1···C6^xii^	3.305 (5)
O3···O10W^x^	3.146 (4)	C2···O2^viii^	3.217 (4)
O3···O9W^viii^	2.821 (3)	C2···C6^xii^	3.426 (5)
O4···O1	3.218 (3)	C2···O9W	3.340 (4)
O4···O5^x^	2.603 (3)	C2···O7^xii^	3.401 (4)
O4···C6^xi^	3.178 (3)	C2···C7^xii^	3.300 (5)
O4···C5^x^	3.336 (3)	C3···C7^xii^	3.304 (5)
O4···O13W^xii^	3.144 (4)	C3···C8^xii^	3.511 (5)
O4···C7^xi^	3.171 (4)	C3···O7^xii^	3.401 (4)
O4···O7^xi^	3.222 (3)	C3···O2^viii^	3.291 (4)
O5···C4^i^	3.335 (3)	C4···O7^xi^	3.252 (4)
O5···O6	3.228 (3)	C4···C5^xii^	3.499 (5)
O5···O4^i^	2.603 (3)	C4···C8^xii^	3.347 (5)
O5···O6^vi^	3.219 (3)	C4···C7^xii^	3.578 (4)
O5···O11W	3.191 (4)	C4···O5^x^	3.335 (3)
O5···O13W	3.015 (3)	C5···O4^i^	3.336 (3)
O6···O13W^xiii^	2.736 (3)	C5···C4^xiii^	3.499 (5)
O6···C5^vi^	3.229 (4)	C5···O6^vi^	3.229 (4)
O6···O11W^vi^	2.777 (3)	C5···C1^xiii^	3.506 (5)
O6···O5	3.228 (3)	C6···O6^vi^	3.238 (4)
O6···C6^vi^	3.238 (4)	C6···O4^xi^	3.178 (3)
O6···O5^vi^	3.219 (3)	C6···C1^xiii^	3.305 (5)
O6···O7	3.229 (3)	C6···C2^xiii^	3.426 (5)
O7···C4^xi^	3.252 (4)	C7···C2^xiii^	3.300 (5)
O7···O1W^vi^	3.193 (3)	C7···C4^xiii^	3.578 (4)
O7···O12W^xi^	2.711 (3)	C7···O8^xi^	3.377 (4)
O7···C3^xiii^	3.401 (4)	C7···C3^xiii^	3.304 (5)
O7···O6	3.229 (3)	C7···O4^xi^	3.171 (4)
O7···O1W^xiv^	3.193 (3)	C8···O1	3.372 (3)
O7···O4^xi^	3.222 (3)	C8···O8^xi^	3.310 (4)
O7···C2^xiii^	3.401 (4)	C8···C3^xiii^	3.511 (5)
O7···C1^xi^	3.266 (4)	C8···C4^xiii^	3.347 (5)
O7···O8	3.215 (3)	C1···H9A	3.02 (4)
O8···C1	3.373 (3)	C1···H8	2.6600
O8···O7	3.215 (3)	C2···H9A	3.06 (4)
O8···O13W^xii^	3.014 (3)	C2···H10B^ii^	2.78 (4)
O8···O1	2.575 (3)	C3···H9B^viii^	2.78 (4)
O8···C7^xi^	3.377 (4)	C5···H4^i^	2.6100
O8···C8^xi^	3.310 (4)	C6···H13A^xiii^	2.93 (4)
O9W···O3^viii^	2.821 (3)	C6···H11B^vi^	2.77 (4)
O9W···C2	3.340 (4)	C7···H12B^xi^	2.76 (4)
O9W···O9W^iii^	3.229 (4)	H1W···H9A^iv^	2.26 (5)
O9W···O10W	3.142 (4)	H1W···O9W^i^	2.74 (3)
O9W···O12W	3.026 (4)	H1W···H9A^i^	2.14 (5)
O9W···O1W^xv^	3.197 (5)	H1W···O2^ii^	2.55 (4)
O9W···O1W^x^	3.197 (5)	H1W···O7^vii^	2.47 (4)
O9W···C1	3.366 (5)	H1W···H11A^iii^	2.31 (4)
O10W···O3^i^	3.146 (4)	H4···C5^x^	2.6100
O10W···O10W^ii^	3.171 (4)	H4···O5^x^	1.7900
O10W···O13W^ii^	3.103 (4)	H8···O1	1.7700
O10W···O9W	3.142 (4)	H8···C1	2.6600
O10W···O11W	3.204 (4)	H9A···O1W^x^	2.47 (3)
O10W···O2^ii^	2.711 (4)	H9A···C1	3.02 (4)
O10W···O11W^iii^	3.150 (4)	H9A···O1W^xv^	2.47 (3)
O11W···O10W^iii^	3.150 (4)	H9A···H1W^xv^	2.26 (5)
O11W···O12W	2.975 (4)	H9A···C2	3.06 (4)
O11W···C3^i^	3.384 (4)	H9A···H1W^x^	2.14 (5)
O11W···O1W	2.880 (5)	H9B···O3^viii^	1.97 (4)
O11W···O5	3.191 (4)	H9B···C3^viii^	2.78 (4)
O11W···O6^vi^	2.777 (3)	H10A···O12W^iii^	2.85 (5)
O11W···O1W	2.880 (5)	H10A···O11W^iii^	2.54 (4)
O11W···O10W	3.204 (4)	H10A···H12A^iii^	2.55 (7)
O12W···O7^xi^	2.711 (3)	H10A···H11B^iii^	2.52 (7)
O12W···O11W	2.975 (4)	H10B···C2^ii^	2.78 (4)
O12W···O9W	3.026 (4)	H10B···O2^ii^	1.88 (4)
O12W···O1	3.104 (4)	H11A···H1W^iii^	2.31 (4)
O12W···O13W^v^	2.803 (3)	H11A···O1W	2.06 (4)
O12W···C5	3.412 (5)	H11A···O1W	2.06 (4)
O13W···O3^i^	3.024 (3)	H11B···C6^vi^	2.77 (4)
O13W···O10W^ii^	3.103 (4)	H11B···O6^vi^	1.91 (4)
O13W···O6^xii^	2.736 (3)	H11B···H10A^iii^	2.52 (7)
O13W···O12W^ix^	2.803 (3)	H12A···H13A^v^	2.36 (6)
O13W···O4^xiii^	3.144 (4)	H12A···O13W^v^	2.00 (4)
O13W···O8^xiii^	3.014 (3)	H12A···H10A^iii^	2.55 (7)
O13W···O1	3.130 (3)	H12A···H13B^v^	2.31 (5)
O13W···O5	3.015 (3)	H12B···O7^xi^	1.87 (4)
O1···H8	1.7700	H12B···C7^xi^	2.76 (4)
O1···H13A	2.74 (4)	H13A···C6^xii^	2.93 (4)
O1···H12B	2.88 (4)	H13A···H12A^ix^	2.36 (6)
O1W···H9A^i^	2.47 (3)	H13A···O4^xiii^	2.68 (5)
O1W···H9A^iv^	2.47 (3)	H13A···O6^xii^	2.00 (4)
O1W···H11A^iii^	2.06 (4)	H13B···H12A^ix^	2.31 (5)
O1W···H11A	2.06 (4)	H13B···O4^xiii^	2.83 (5)
O2···H10B^ii^	1.88 (4)	H13B···O8^xiii^	2.24 (4)
O2···H1W^ii^	2.55 (4)		
			
O1—Ba1—O5	82.21 (6)	O12W—Sr1—O13W	127.59 (10)
O1—Ba1—O9W	74.79 (8)	O3^i^—Sr1—O12W	138.07 (7)
O1—Ba1—O10W	140.69 (7)	O10W^ii^—Sr1—O12W	138.54 (8)
O1—Ba1—O11W	134.79 (7)	O3^i^—Sr1—O13W	67.32 (7)
O1—Ba1—O12W	70.57 (8)	O10W^ii^—Sr1—O13W	67.73 (8)
O1—Ba1—O13W	69.21 (6)	O3^i^—Sr1—O10W^ii^	82.41 (7)
O1—Ba1—O3^i^	136.38 (8)	Ba1—O1—C1	129.4 (2)
O1—Ba1—O10W^ii^	84.71 (7)	Sr1—O1—C1	129.4 (2)
O5—Ba1—O9W	142.14 (7)	Ba1^x^—O3—C3	134.23 (18)
O5—Ba1—O10W	136.94 (7)	Ba1—O5—C5	130.74 (18)
O5—Ba1—O11W	72.29 (8)	Sr1—O5—C5	130.74 (18)
O5—Ba1—O12W	75.68 (8)	Ba1—O10W—Ba1^ii^	110.43 (10)
O5—Ba1—O13W	66.88 (7)	C4—O4—H4	109.00
O3^i^—Ba1—O5	77.84 (6)	C8—O8—H8	109.00
O5—Ba1—O10W^ii^	134.51 (8)	Ba1—O9W—H9A	120 (3)
O9W—Ba1—O10W	69.68 (9)	H9A—O9W—H9B	115 (4)
O9W—Ba1—O11W	103.67 (9)	Sr1—O9W—H9B	117 (3)
O9W—Ba1—O12W	68.52 (9)	Ba1—O9W—H9B	117 (3)
O9W—Ba1—O13W	128.28 (8)	Sr1—O9W—H9A	120 (3)
O3^i^—Ba1—O9W	138.55 (8)	Ba1—O10W—H10B	113 (3)
O9W—Ba1—O10W^ii^	73.22 (8)	Ba1—O10W—H10A	108 (3)
O10W—Ba1—O11W	71.37 (8)	Sr1—O10W—H10A	108 (3)
O10W—Ba1—O12W	109.89 (10)	Sr1—O10W—H10B	113 (3)
O10W—Ba1—O13W	122.53 (8)	H10A—O10W—H10B	100 (5)
O3^i^—Ba1—O10W	70.58 (8)	Ba1^ii^—O10W—H10A	115 (3)
O10W—Ba1—O10W^ii^	69.57 (8)	Ba1^ii^—O10W—H10B	110 (2)
O11W—Ba1—O12W	67.33 (8)	Ba1—O11W—H11A	123 (2)
O11W—Ba1—O13W	128.05 (8)	Ba1—O11W—H11B	115 (3)
O3^i^—Ba1—O11W	73.96 (7)	H11A—O11W—H11B	114 (4)
O10W^ii^—Ba1—O11W	139.20 (8)	Sr1—O11W—H11A	123 (2)
O12W—Ba1—O13W	127.59 (10)	Sr1—O11W—H11B	115 (3)
O3^i^—Ba1—O12W	138.07 (7)	H12A—O12W—H12B	110 (4)
O10W^ii^—Ba1—O12W	138.54 (8)	Ba1—O12W—H12A	128 (3)
O3^i^—Ba1—O13W	67.32 (7)	Ba1—O12W—H12B	118 (3)
O10W^ii^—Ba1—O13W	67.73 (8)	Sr1—O12W—H12A	128 (3)
O3^i^—Ba1—O10W^ii^	82.41 (7)	Sr1—O12W—H12B	118 (3)
O1—Sr1—O5	82.21 (6)	Ba1—O13W—H13A	110 (3)
O1—Sr1—O9W	74.79 (8)	Ba1—O13W—H13B	109 (3)
O1—Sr1—O10W	140.69 (7)	Sr1—O13W—H13A	110 (3)
O1—Sr1—O11W	134.79 (7)	Sr1—O13W—H13B	109 (3)
O1—Sr1—O12W	70.57 (8)	H13A—O13W—H13B	111 (4)
O1—Sr1—O13W	69.21 (6)	H1W—O1W—H1W^iii^	111 (3)
O1—Sr1—O3^i^	136.38 (8)	C2—C1—C4	89.3 (2)
O1—Sr1—O10W^ii^	84.71 (7)	O1—C1—C2	133.6 (2)
O5—Sr1—O9W	142.14 (7)	O1—C1—C4	137.1 (3)
O5—Sr1—O10W	136.94 (7)	C1—C2—C3	88.6 (2)
O5—Sr1—O11W	72.29 (8)	O2—C2—C1	136.0 (3)
O5—Sr1—O12W	75.68 (8)	O2—C2—C3	135.3 (3)
O5—Sr1—O13W	66.88 (7)	O3—C3—C4	136.1 (3)
O3^i^—Sr1—O5	77.84 (6)	O3—C3—C2	135.3 (3)
O5—Sr1—O10W^ii^	134.51 (8)	C2—C3—C4	88.6 (2)
O9W—Sr1—O10W	69.68 (9)	O4—C4—C3	135.1 (2)
O9W—Sr1—O11W	103.67 (9)	O4—C4—C1	131.4 (2)
O9W—Sr1—O12W	68.52 (9)	C1—C4—C3	93.4 (2)
O9W—Sr1—O13W	128.28 (8)	C6—C5—C8	89.9 (2)
O3^i^—Sr1—O9W	138.55 (8)	O5—C5—C6	133.7 (2)
O9W—Sr1—O10W^ii^	73.22 (8)	O5—C5—C8	136.4 (3)
O10W—Sr1—O11W	71.37 (8)	C5—C6—C7	89.2 (2)
O10W—Sr1—O12W	109.89 (10)	O6—C6—C5	135.6 (3)
O10W—Sr1—O13W	122.53 (8)	O6—C6—C7	135.1 (3)
O3^i^—Sr1—O10W	70.58 (8)	C6—C7—C8	88.1 (2)
O10W—Sr1—O10W^ii^	69.57 (8)	O7—C7—C6	134.7 (3)
O11W—Sr1—O12W	67.33 (8)	O7—C7—C8	137.1 (3)
O11W—Sr1—O13W	128.05 (8)	C5—C8—C7	92.8 (2)
O3^i^—Sr1—O11W	73.96 (7)	O8—C8—C5	136.6 (3)
O10W^ii^—Sr1—O11W	139.20 (8)	O8—C8—C7	130.5 (3)
			
O5—Sr1—O1—C1	−179.3 (2)	O1—C1—C2—C3	−178.6 (4)
O9W—Sr1—O1—C1	−29.6 (2)	C4—C1—C2—O2	176.0 (4)
O10W—Sr1—O1—C1	−3.8 (3)	O2—C2—C3—O3	1.7 (7)
O11W—Sr1—O1—C1	−123.9 (2)	C1—C2—C3—C4	1.2 (3)
O12W—Sr1—O1—C1	−101.8 (3)	O2—C2—C3—C4	−176.0 (4)
O13W—Sr1—O1—C1	112.6 (2)	C1—C2—C3—O3	178.8 (4)
O3^i^—Sr1—O1—C1	117.6 (2)	O3—C3—C4—O4	−2.4 (7)
O10W^ii^—Sr1—O1—C1	44.4 (2)	C2—C3—C4—C1	−1.2 (3)
O1—Sr1—O5—C5	36.9 (2)	O3—C3—C4—C1	−178.9 (4)
O9W—Sr1—O5—C5	−15.7 (3)	C2—C3—C4—O4	175.3 (4)
O10W—Sr1—O5—C5	−138.9 (2)	O5—C5—C6—O6	1.2 (7)
O11W—Sr1—O5—C5	−105.3 (3)	O5—C5—C6—C7	177.8 (4)
O12W—Sr1—O5—C5	−34.9 (3)	C8—C5—C6—O6	−176.1 (4)
O13W—Sr1—O5—C5	107.6 (3)	C8—C5—C6—C7	0.5 (3)
O3^i^—Sr1—O5—C5	177.9 (3)	O5—C5—C8—O8	−2.2 (7)
O10W^ii^—Sr1—O5—C5	111.5 (3)	O5—C5—C8—C7	−177.7 (4)
Sr1—O1—C1—C2	−32.4 (5)	C6—C5—C8—O8	174.9 (4)
Sr1—O1—C1—C4	151.4 (3)	C6—C5—C8—C7	−0.6 (3)
Sr1—O5—C5—C6	156.2 (3)	O6—C6—C7—O7	−1.1 (7)
Sr1—O5—C5—C8	−27.8 (6)	O6—C6—C7—C8	176.1 (4)
O1—C1—C2—O2	−1.4 (7)	C5—C6—C7—O7	−177.8 (4)
C4—C1—C2—C3	−1.2 (3)	C5—C6—C7—C8	−0.5 (3)
O1—C1—C4—O4	1.8 (7)	O7—C7—C8—O8	1.7 (7)
O1—C1—C4—C3	178.4 (4)	O7—C7—C8—C5	177.7 (4)
C2—C1—C4—O4	−175.5 (4)	C6—C7—C8—O8	−175.4 (4)
C2—C1—C4—C3	1.2 (3)	C6—C7—C8—C5	0.6 (3)

Symmetry codes: (i) *x*, *y*−1, *z*; (ii) −*x*+1, −*y*, −*z*+1; (iii) −*x*+1, *y*, −*z*+1/2; (iv) −*x*+1, *y*−1, −*z*+1/2; (v) *x*, −*y*, *z*−1/2; (vi) −*x*+1/2, −*y*−1/2, −*z*; (vii) *x*+1/2, −*y*−1/2, *z*+1/2; (viii) −*x*+1, −*y*+1, −*z*+1; (ix) *x*, −*y*, *z*+1/2; (x) *x*, *y*+1, *z*; (xi) −*x*+1/2, −*y*+1/2, −*z*; (xii) −*x*+1/2, *y*+1/2, −*z*+1/2; (xiii) −*x*+1/2, *y*−1/2, −*z*+1/2; (xiv) *x*−1/2, −*y*−1/2, *z*−1/2; (xv) −*x*+1, *y*+1, −*z*+1/2.

### Hydrogen-bond geometry (Å, °)

**Table:**

*D*—H···*A*	*D*—H	H···*A*	*D*···*A*	*D*—H···*A*
O1W—H1W···O2^ii^	0.82 (4)	2.55 (4)	2.874 (2)	105 (3)
O1W—H1W···O7^vii^	0.82 (4)	2.47 (4)	3.193 (3)	148 (4)
O4—H4···O5^x^	0.82	1.79	2.603 (3)	171.
O8—H8···O1	0.82	1.77	2.575 (3)	169.
O9W—H9A···O1W^x^	0.84 (3)	2.47 (3)	3.197 (5)	147 (4)
O9W—H9B···O3^viii^	0.87 (4)	1.97 (4)	2.821 (3)	167 (5)
O10W—H10A···O11W^iii^	0.78 (5)	2.54 (4)	3.150 (4)	137 (4)
O10W—H10B···O2^ii^	0.86 (4)	1.88 (4)	2.711 (4)	164 (4)
O11W—H11A···O1W	0.86 (3)	2.06 (4)	2.880 (5)	159 (4)
O11W—H11B···O6^vi^	0.87 (4)	1.91 (4)	2.777 (3)	176 (5)
O12W—H12A···O13W^v^	0.83 (4)	2.00 (4)	2.803 (3)	164 (4)
O12W—H12B···O7^xi^	0.84 (4)	1.87 (4)	2.711 (3)	178 (5)
O13W—H13A···O6^xii^	0.86 (5)	2.00 (4)	2.736 (3)	143 (3)
O13W—H13B···O8^xiii^	0.84 (4)	2.24 (4)	3.014 (3)	154 (4)

Symmetry codes: (ii) −*x*+1, −*y*, −*z*+1; (vii) *x*+1/2, −*y*−1/2, *z*+1/2; (x) *x*, *y*+1, *z*; (viii) −*x*+1, −*y*+1, −*z*+1; (iii) −*x*+1, *y*, −*z*+1/2; (vi) −*x*+1/2, −*y*−1/2, −*z*; (v) *x*, −*y*, *z*−1/2; (xi) −*x*+1/2, −*y*+1/2, −*z*; (xii) −*x*+1/2, *y*+1/2, −*z*+1/2; (xiii) −*x*+1/2, *y*−1/2, −*z*+1/2.
